# Immunopathophysiology of human sepsis

**DOI:** 10.1016/j.ebiom.2022.104363

**Published:** 2022-12-02

**Authors:** W. Joost Wiersinga, Tom van der Poll

**Affiliations:** Amsterdam University Medical Centres, University of Amsterdam, Center of Experimental and Molecular Medicine & Division of Infectious Diseases, Amsterdam, the Netherlands

**Keywords:** Sepsis, Pathogenesis, Hyperinflammation, Immune suppression

## Abstract

Sepsis is an ill-defined syndrome yet is a leading cause of morbidity and mortality worldwide. The most recent consensus defines sepsis as life-threatening organ dysfunction caused by a dysregulated host response to infection. However, this definition belies the complexity and breadth of immune mechanisms involved in sepsis, which are characterized by simultaneous hyperinflammation and immune suppression. In this review, we describe the immunopathogenesis of sepsis and highlight some recent pathophysiological findings that have expanded our understanding of sepsis. Sepsis endotypes can be used to divide sepsis patients in different groups with distinct immune profiles and outcomes. We also summarize evidence on the role of the gut microbiome in sepsis immunity. The challenge of the coming years will be to translate our increasing knowledge about the molecular mechanisms underlying sepsis into therapies that improve relevant patient outcomes.

## Introduction

Sepsis is a dysregulated host response to infection leading to fatal organ and tissue injury.^1^^,^^2^ Sepsis manifests heterogeneously and symptoms are usually specific to the source of infection.^3^ Sepsis is one of the leading global causes of mortality with 11 million deaths in 2017 and is expected to become a greater issue due to aging populations with suppressed immunity, advances in medical care with associated immune modulating medications and global warming.^4^

The pathogenesis of sepsis is complex beyond the type of infection and its initial host response, and involves heterogeneous features of inflammation, activation of coagulation, the vascular endothelium and the complement system, immune suppression, and alterations in the microbiome.^2^ Inflammation is initiated to eradicate an infectious pathogen and varies in intensity and scope according to type, load and virulence of the pathogen, patient comorbidities, immune fitness, sex, age and nutritional status.^2^ Anti-inflammatory responses regulate inflammation, facilitate repair mechanisms and contribute to homeostasis return.^5^ However, unbalanced anti-inflammatory reactions can result in sustained immunosuppression, which can make the patient susceptible to secondary infections.^5^ Sepsis patients requiring intensive care for prolonged periods of time often develop a chronic critical illness named “persistent inflammation, immunosuppression and catabolism syndrome” (PICS), characterized by protracted hyperinflammation, immune suppression, dysregulated myelopoiesis and catabolism.^6^

After decades of research sepsis remains ill-defined, and the current Sepsis-3 definition does not fully capture the complexity of the syndrome.^1^ Whilst our knowledge on the pathogenesis of sepsis has increased considerably, many questions remain and the translation to specific sepsis therapies has proved to be difficult. At present, the treatment of sepsis continues to be supportive with interventions centred on antibiotics, resuscitation and support of organ dysfunction.^7^ Recent attempts have focussed on grouping patients with sepsis into more homogenous subgroups based on measurable clinical and pathobiological characteristics with possible relevance for treatment decisions.^2^^,^^8^

Here we review the current understanding of the pathogenesis of sepsis and highlight recent findings in this field.

## Pathogens

Any infection can result in sepsis, irrespective of the type of pathogen. Whilst bacteria, fungi, parasites or viruses can cause sepsis, bacteria are most commonly identified.^3^^,^^9^ Viruses are less often considered causative in sepsis, although their burden could be underestimated in paediatric sepsis and the tropics.^10^^,^^11^ Fungal sepsis is increasing in incidence and is associated with a high mortality.^9^^,^^12^ In 30–50% of sepsis cases the causative pathogen remains unknown or unclear.^3^^,^^9^ In the western world infections of the respiratory tract are the most common cause of sepsis, accounting for up to 50% of cases; other sources include the abdomen, urinary tract, skin or central nervous system.^3^^,^^9^ Infections acquired during hospitalization are common. Intubation and catheters can provide entry for exogenous pathogens, which together with suppressed immunity resulting from the condition that necessitated hospital admission can create fertile ground for hospital-acquired pneumonia and other nosocomial infections.^9^^,^^13^

While viruses, parasites, fungi and bacteria vastly differ in structure, virulence and infection mechanisms, they all contain pathogen-associated molecular patterns (PAMPs).^3^^,^^14^^,^^15^ PAMPs are conserved motifs expressed by microbes that—depending on pathogen type—can be of various compositions. Well-studied PAMPs in the setting of sepsis include bacterial lipopolysaccharide (LPS), flagellin and lipoteichoic acid, and viral RNA and DNA (Table 1).^14^

**Table 1:** Examples of key PAMPs and DAMPs in sepsis pathophysiology.

	Origin	PRR
**PAMPs**		
(Diacyl/triacyl) lipopetides	Gram-positive/Gram-negative bacteria	TLR1,TLR2,TLR6
LTA	Gram-positive bacteria	TLR2
Peptidoglycan	Gram-positive/Gram-negative bacteria	TLR2
dsRNA	Double-stranded RNA virus	TLR3, RIG-1
LPS	Gram-negative bacteria	TLR4
Flagellin	Gram-positive/Gram-negative bacteria	TLR5
ssRNA	Singe-stranded RNA virus	TLR7, TLR8
CpG DNA	Bacteria	TLR9
**DAMPs**		
HMGB-1	Nucleus, autophagosome	TLR2, TLR4, TLR9, RAGE
Histone	Nucleus	TLR2, TLR4, NLRP3
dsDNA	Cytosol	RIG-1, MDA5, STING
S100A8/A9	Cytosol	TLR4, RAGE
Heat shock proteins	Cytosol, mitochondria, nucleus	TLR2, TLR4, CLR LOX-1
Heparan sulfate	Extracellular matrix component	TLR4
Tenascin-C	Extracellular matrix component	TLR4
Oxidized LPL	Triglycerides	TLR4

Abbreviations: CLR: C-type lectin receptors; DAMPs: damage-associated molecular patterns; HMGB1: high-mobility group protein B1; LOX-1: low-density lipoprotein receptor-1; LTA: lipoteichoic acid; LPL: lipoprotein lipase; LPS: lipopolysaccharide; MDA5: melanoma differentiation-associated protein 5; PAMPs: pathogen-associated molecular patterns; PRR: pattern-recognition receptor; RIG-I: retinoic acid-inducible gene I; STING: stimulator of interferon genes.

Of note, only bacterial PAMPs are listed and only the primary PRRs of the indicated PAMPs or DAMPs.

## Pattern recognition

PAMPs make pathogens recognizable.^15^ The immune system detects invading pathogens’ PAMPs through pattern recognition receptors (PRRs), which are expressed by many different immune and parenchymal cells.^15^ Prominent PRR families include Toll-like receptors (TLRs), nucleotide-binding oligomerization domain-like receptors (NOD)-like receptor (NLRs), C-type lectin receptors (CLRs), retinoic acid-inducible gene (RIG)-I-like receptors (RLRs), and cytosolic RNA and DNA sensors.^14^^,^^16^

Upon recognizing and interacting with PAMPs, PRRs upregulate inflammatory genes and initiate innate immunity to battle the pathogen. Innate immunity is the first line of defence against invading pathogens and the initial encounter between pathogen and host results in localized pro-inflammatory, anti-inflammatory and reparative responses aimed at eradication of the invading microorganism and a return to homeostasis. When PAMPs are detected, PRRs activate signalling pathways that coordinate inflammation and initiate the secretion of inflammatory cytokines and other molecules.^15^ If the host response to a multiplying pathogen is successful, a balanced immune response ensues that clears the infection. However, in some instances pathogens can evade immune defence mechanisms, and the growing pathogen load can overwhelm already activated PRRs and knock the host response off balance.^2^ As a consequence, PRRs are persistently stimulated and protective inflammatory and anti-inflammatory mechanisms become destructive, leading to tissue injury and sepsis.^2^ PRRs not only recognize exogenous PAMPs, but also interact with endogenous alarmins, or damage-associated molecular patterns (DAMPs). DAMPs are released by injured host cells,^17^ and can be proteins, such as heat shock proteins and high mobility group box (HMGB)-1, DNA or RNA, or non-protein metabolites such as uric acid and ATP.^17^^,^^18^ DAMPs have been implicated in a vicious cycle of cell injury and sustained hyperinflammation (Table 1).^17^

## Hyperinflammation

**Fig. 1:**
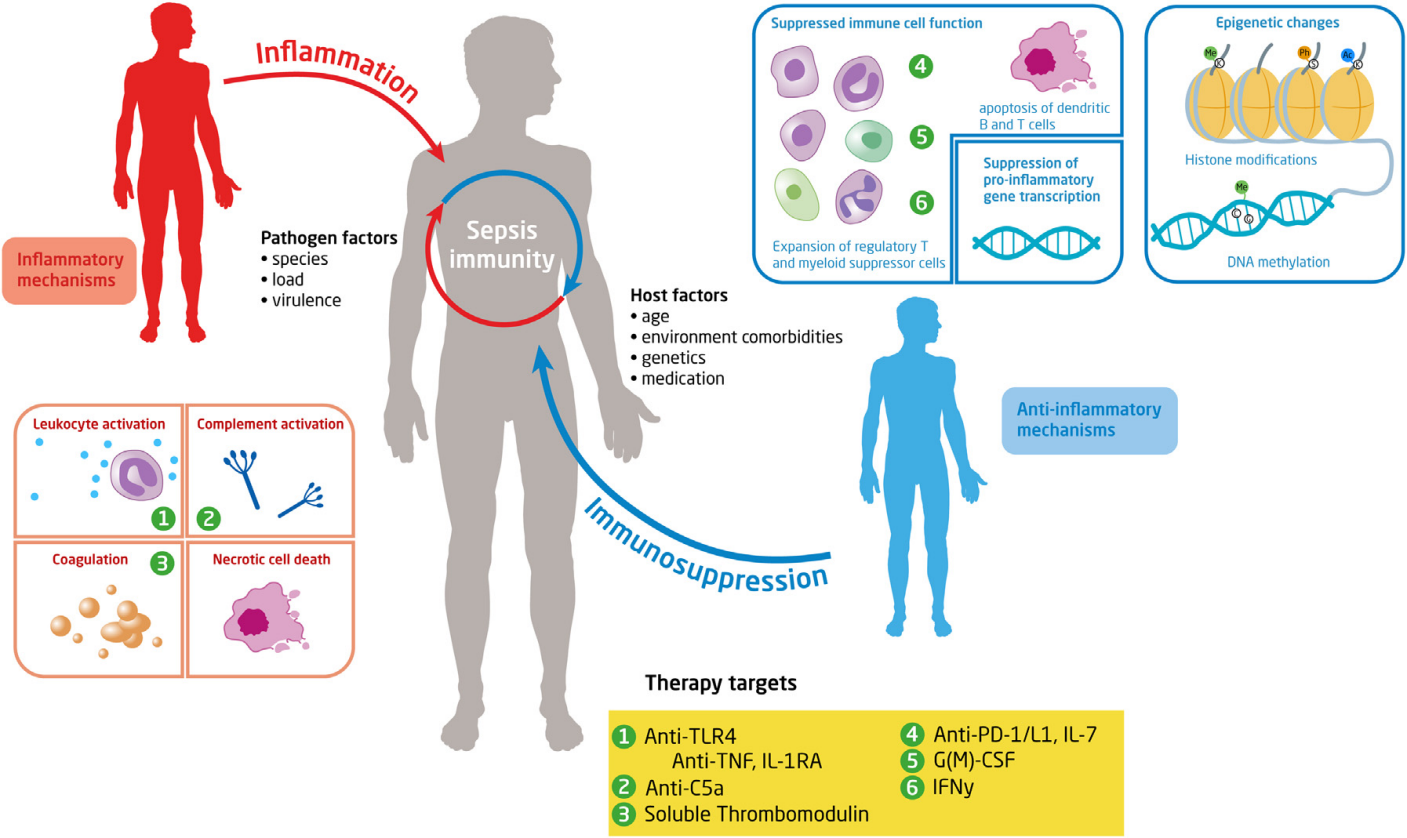
**Sepsis immunity and therapeutic targets.** Sepsis is characterized by the simultaneous interplay of pro- and anti-inflammatory mechanisms. The magnitude of the immune response in sepsis depends on pathogen and host related factors. The proinflammatory response is characterized by among others the release of pro-inflammatory mediators, activation of the complement and the coagulation systems and the release of alarmins by necrotic cell death. Excessive inflammation can cause collateral damage to healthy tissue. The anti-inflammatory response is characterized by impaired immune cell function due to effector cell apoptosis, T cell exhaustion, reduced monocyte HLA-DR expression, increased expression of suppressor cells and inhibition of pro-inflammatory gene transcription. Anti-inflammatory changes may relate to epigenetic changes, in particular histone modifications and alterations in DNA methylation. Histones determine the accessibility of DNA to transcription factors; histone function can be modified by acetylation (Ac), methylation (Me), and phosphorylation (Ph) of their tails. DNA methylation happens at cytosine-guanine dinucleotides (denoted by C and G). The yellow boxes indicate a selection of therapeutic targets that have been clinically evaluated as interventions in the septic immune response. Abbreviations: TLR4: Toll-like receptor-4; TNF: tumour necrosis factor; PD-1: programmed cell death protein; IL-1RA: interleukin-1 receptor antagonist; IFN: interferon; G(M)-CSF: Granulocyte macrophage-colony stimulating factor.

Hyperinflammation in sepsis manifests as shock and fever. Inflammation in sepsis is mainly driven by leukocytes, cytokines, oxygen radicals, endothelial cells, and the complement and coagulation systems (Fig. 1). Whilst local activation of these proinflammatory and procoagulant mechanisms after infection is part of protective innate immunity, their uncontrolled activity causes collateral damage and plays a key role in the pathogenesis of sepsis. Uncontrolled activity of pro-inflammatory cytokines such as tumour necrosis factor (TNF) and IL-1β is considered to play an important role in tissue injury.^19^ Inflammation in sepsis is amplified by activated neutrophils, which release reactive oxygen species and proteases. Proteases can be part of neutrophil extracellular traps (NETs), which are extracellular fibre networks released by activated neutrophils, and that besides proteases, contain histones and DNA.^20^ Disruption of NETs increased bacterial burdens and mortality in experimental sepsis, demonstrating the importance of NETs in host defence. On the other hand, excessive NETosis during sepsis can cause tissue damage and overdrive the coagulation system, thereby contributing to intravascular thrombosis, disseminated intravascular coagulation and organ failure.^21^ NET-embedded histones can attach to and activate the endothelium, resulting in vascular damage.^22^ As another contributor to the vicious septic cycle, evidence increasingly points to cross-talk between DAMPs and NETs. NETs are composed of DAMPs (e.g., histones and DNA) that can activate PRRs,^17^ while DAMPs can trigger NETosis; for example, HMBG-1 promotes NET formation via TLR4.^23^

Crucial proinflammatory pathways implicated in the pathogenesis of sepsis are the complement and coagulation systems. The complement system can be activated by various agonists, including PAMPs and DAMPs, and marks intruders for lysis and opsonization.^24^^,^^25^ Activation of the complement system is associated with the release of C3a and C5a, which are powerful proinflammatory molecules that recruit and activate immune cells and platelets.^24^ C3a and C5a also regulate vascular flow, increase vascular permeability and promote leukocyte adhesion and mobility.^26^ Yet, unrestrained complement activation can injure tissues and organs.^24^ Indeed, C5a elimination improved the outcome in several sepsis models,^27^ and the C3 convertase inhibitor compstatin not only attenuated complement activation during *Escherichia coli* sepsis in baboons, but also inhibited other inflammatory responses, activation of coagulation and multiple organ failure.^28^

Sepsis is associated with a disbalance in the coagulation system, characterized by uncontrolled procoagulant activity with concurrent impairment of endogenous anticoagulant mechanisms.^29^ Disseminated intravascular coagulation is a severe manifestation of unbalanced coagulation, clinically presenting as thrombosis and haemorrhage due to consumption of clotting factors, anticoagulant proteins and platelets.^29^ Tissue factor (TF) is considered the main driver of coagulation activation.^30^ Microbial agents, cytokines and complements factors trigger the production of TF by macrophages and monocytes. Moreover, TF can be detected in microvesicles in the circulation of sepsis patients, which originate from several cellular sources and can amplify coagulation. Blockade of tissue factor in humans and non-human primates strongly reduced coagulation activation during experimental endotoxemia and bacteraemia, and in septic baboons prevented multiple organ failure and mortality.^29^ Besides by excessive coagulation, physiologic haemostasis is disrupted in sepsis due to diminished function of regulatory anticoagulant pathways: antithrombin, tissue factor pathway inhibitor and the protein C system. These coagulation inhibitors are reduced in sepsis, creating fertile ground for excess coagulation activation.^29^ Reduction of the anticoagulant capacity is for a large part due to inflammation-induced damage to the glycocalyx, a glycoprotein-polysaccharide layer at the surface of the vascular endothelium. Moreover, enhanced production of nitric oxide and prostanoids impacts vascular homeostasis and a variety of inflammatory processes.

Hyperinflammation in sepsis is aggravated by tight interactions between different mediator systems. Clotting factors can activate complement and vice versa, with thrombin-induced formation of C3a and C5a, and C5a mediated expression of TF on endothelial cells as illustrative examples.^31^ Moreover, many clotting factors can initiate inflammatory responses through activation of protease activated receptors.^32^ NETs stimulate platelet activation and aggregation, while in turn activated platelets attract and activate neutrophils, reducing their threshold for the formation of NETs. In experimental sepsis platelet activation and fibrin formation can be detected in NETs, which is important for the development of disseminated intravascular coagulation.^21^ Neutrophil derived proteases can enhance thrombus growth in vivo by degrading tissue factor pathway inhibitor (TFPI).^33^ Recent evidence suggests a role for gasdermin D in the connection between inflammation and coagulation.^34^ Specifically, gasdermin D, upon cleavage by caspase-1 or -11, can induce pyroptosis, a proinflammatory form of lytic programmed cell death, thereby permitting the release of TF containing microvesicles.

## Immune suppression

Anti-inflammatory mechanisms are triggered to regulate inflammation, prevent autoimmunity and guide the host to homeostasis.^5^ However, in sepsis anti-inflammatory reactions can become unbalanced and result in sustained immune suppression. Sepsis-associated immune suppression involves many different cell types and is characterized by immune cell depletion, cellular apoptosis, T cell exhaustion, reprogramming of antigen-presenting cells, among others featured by a diminished capacity to produce pro-inflammatory cytokines upon stimulation and reduced expression of cell-surface molecules such as HLA-DR.^5^ Lack of these crucial effector functions can lead to a failure in eradicating the primary infection, render patients susceptible for secondary opportunistic infections and reactivate dormant viruses.^5^^,^^35^^,^^36^

Apoptosis in sepsis reduces the host's repertoire of effector immune cells. CD4^+^ and CD8^+^ T cells, B cells, natural killer cells and dendritic cells are especially depleted in sepsis.^5^ Apoptosis has been implicated as an important factor in sepsis-associated mortality considering that interventions that inhibit apoptosis improved survival in animal sepsis models. Lymphopenia has been used as an easily measurable indicator of immune suppression; lymphopenia in sepsis may not only be due to enhanced apoptosis: other mechanisms include enhanced extravasation and migration to sites of inflammation. B lymphocytes in sepsis display an exhausted phenotype, with reduced major histocompatibility complex class 2 (MHC II) expression and increased IL-10 production, an anti-inflammatory cytokine.^37^

Sepsis-induced immune suppression is associated with a reprogramming of monocytes and macrophages. The term “endotoxin tolerance” relates to the impaired ability of cells to produce proinflammatory cytokines upon re-stimulation with a bacterial agonist following an initial bacterial challenge.^38^ “Tolerance” does not fully capture the functional change in monocytes and macrophages in sepsis: whilst monocytes of patients with sepsis demonstrate reduced pro-inflammatory cytokine release upon re-stimulation, their ability to produce anti-inflammatory mediators such as IL-1 receptor antagonist and IL-10 is either unaltered or even enhanced.^38^ The reprogramming of monocytes in sepsis is at least in part mediated by epigenetic regulation involving histone modifications and altered DNA methylation. Diminished pro-inflammatory responses upon re-stimulation of blood leukocytes may relate to a reduced capacity to activate nuclear factor-κB, as pointed out by intracellular flow cytometry of ex vivo stimulated monocytes, CD4^+^ T cells, CD8^+^ T cells, B cells and neutrophils from patients with sepsis.^39^ Organ-specific monocytes harvested from sepsis patients shortly after their death also showed an anti-inflammatory phenotype,^40^ although investigations in mice reported a primed state of alveolar macrophages, Kupffer cells, and microglial cells.^41^ Likewise, human alveolar macrophages were primed after an in vivo challenge with LPS,^42^ contrasting with the tolerant state of blood monocytes after intravenous LPS administration.^43^

**Fig. 2:**
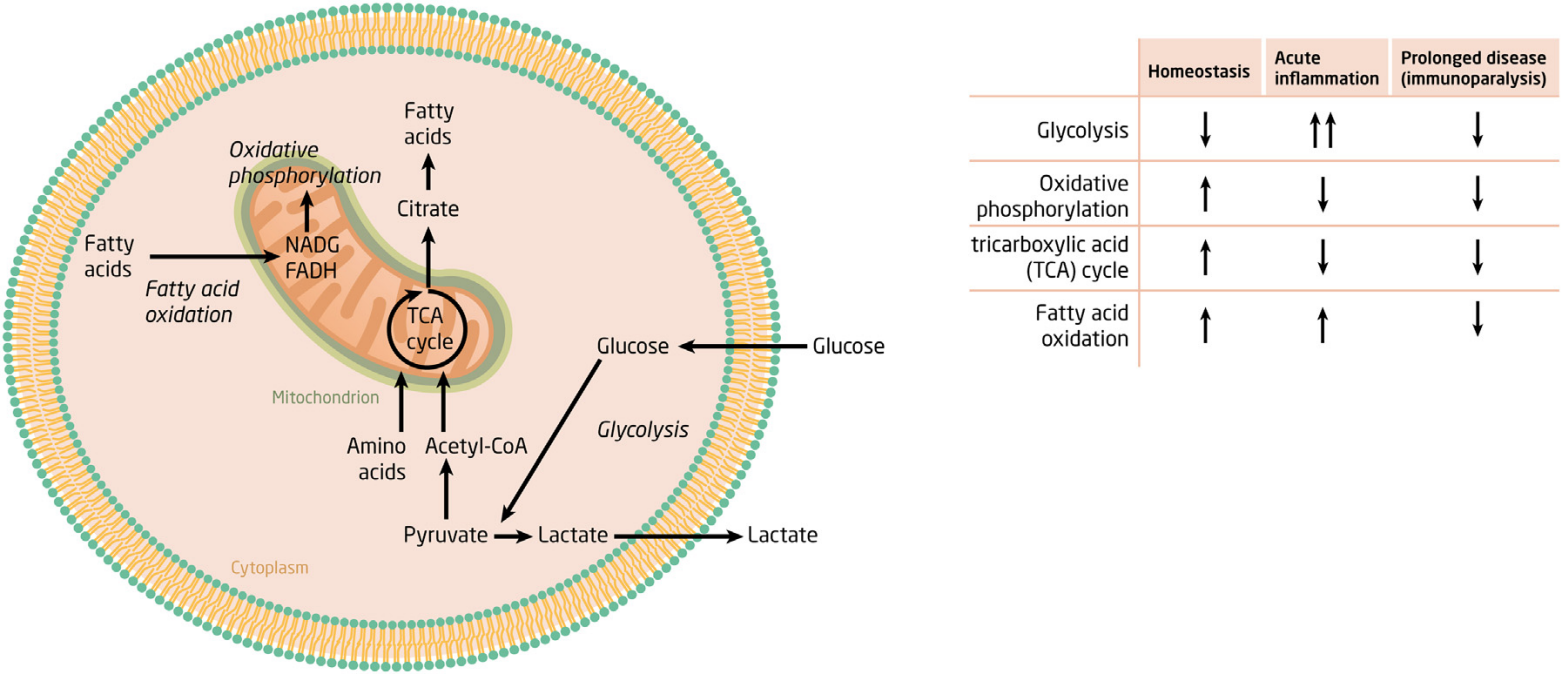
**Immunometabolism in sepsis.** Overview of key cellular energy pathways in innate immune cells in resting state (homeostasis), during acute inflammation and during prolonged critical illness. Arrows indicate upregulation (predominant role) or downregulation.

The function of immune cells depends on their energy state (Fig. 2).^44^ In quiescent conditions, immune cells mainly utilize the tricarboxylic acid (TCA) cycle and oxidative phosphorylation to generate energy, processes that take place in the mitochondria. Glycolysis (the breakdown of glucose to pyruvate and lactate) becomes a major energy pathway in cells upon activation, generating ATP less efficiently yet much faster. Notably, peripheral blood mononuclear cells from sepsis patients displayed a reduced cytokine production capacity (a characteristic feature of immune suppression) together with broad metabolic defects as indicated by reduced ATP and NAD+ content, reduced lactate production and reduced oxygen consumption, a condition termed “immunometabolic paralysis”.^43^

A relative increase in the number of regulatory T (Treg) cells and myeloid-derived suppressor cells (MDSCs) further contributes to immune suppression in sepsis. MDSCs can inhibit immune functions by a variety of mechanisms that include suppression of lymphocyte, macrophage and dendritic cell functions.^45^ Expansion of MDSCs has been associated with a higher frequency of secondary infections in patients with sepsis.^46^^,^^47^ A recent study used single cell RNA sequencing to reveal a CD14+ monocyte phenotype named MS1, characterized by high expression of *RETN*, *ALOX5AP*, and *IL1R2*, that was unique in patients with sepsis and associated with sepsis-induced immunosuppression.^48^ MS1 cells may represent monocytic MDSCs. In co-culture systems MS1 cells suppressed T cell proliferation and inhibited endothelial cell activation, signifying their immune suppressive features.^49^ Neutrophils display a number of immune compromised features in sepsis, including reduced migration to a variety of chemoattractant, lower intracellular myeloperoxidase and lactoferrin content, and reduced oxidative burst capacity.^50^ Kinome profiling revealed lessened kinase activity in neutrophils from patients with sepsis relative to critically ill patients without infection, further indicating an immune suppressed neutrophil phenotype.^51^

Checkpoint regulators direct the immune response to a specific antigen by acting as a second signal.^52^ A checkpoint regulator that has received much attention in the context of sepsis is programmed cell death-1 (PD-1). Sepsis patients demonstrate increased PD-1 expression on T cells, monocytes and granulocytes.^53^^,^^54^ Several investigations have pointed at the functional relevance of the PD-1 pathway in sepsis. Elevated T cell PD-1 levels were associated with impaired T-cell proliferative capacity, a higher frequency of nosocomial infections and increased mortality in patients with sepsis.^53^ PD-1 expression on T cells and PD ligand-1 (PD-L1) expression on antigen presenting cells associated with lymphopenia, T cell apoptosis and mortality in sepsis patients.^40^^,^^53^ Enhanced PD-L1 expression on monocytes and neutrophils correlated with a compromised phagocytosis ability and ex vivo treatment with an anti-PD-1 antibody increased the phagocytic capacity of blood leukocytes from sepsis patients.^55^ The functional relevance of the PD-1 pathway is illustrated by lower mortality rates of septic mice with blocked or genetically eliminated PD-1.^56^^,^^57^

Sepsis-induced immune suppression has been targeted in several trials testing stimulatory therapy, including an anti-PD-L1 antibody and cytokines (reviewed in 5). With regard to recombinant cytokines, IL-7 especially is attractive considering its ability to affect many different immune effector cells implicated in pathogen elimination (e.g., CD4 and CD8 T cells, and innate lymphoid cells) and to improve outcome in experimental sepsis. Notably, administration of IL-7 was well-tolerated in a phase II trial in sepsis patients, and reversed sepsis-induced lymphopenia and enhanced T-cell activation.

An interesting area for future research is the role of sepsis associated encephalopathy in suppression of immune responses in the periphery. The central nervous system (CNS) participates in the regulation of the immune response.^58^ The brain is vulnerable to damage in sepsis, mediated by inflammatory and oxidative processes, which can result in sepsis-associated encephalopathy and a disruption of the physiological interactions between the CNS and the immune system. Animal studies with different types of brain injury have linked brain dysfunction to a variety of immune suppressive effects in the periphery, involving monocytes/macrophages (amongst others increased IL-10 production and M2 type polarization), dendritic cells (reduced responsiveness to TLR stimulation), neutrophils (impaired phagocytosis and reactive oxygen species production) and T lymphocytes (imbalance between Treg cells and proinflammatory lymphocyte subsets).^59^

## Microbiome

The microbial communities that reside in the gut live in symbiosis with the host and play key roles in the development and maturation of the immune system, and the protection against invading pathogens.^60^^,^^61^ The gut microbiome of virtually all critically ill patients with sepsis is severely disrupted.^62^^,^^63^ A decrease in bacterial diversity is seen with a lower relative abundance of *Firmicutes* and *Bacteroidetes* with decreased numbers of obligatory anaerobic gut bacteria, such as *Faecalibacterium prausnitzii* and *Blautia* and *Ruminococcus spp*.^62^^,^^63^ The loss of the anaerobic intestinal environment is directly correlated with an overgrowth of aerobic pathobionts such as *Enterobacter*, *Enterococcus* and *Staphylococcus*, and their corresponding bacteriophages, as well as an absolute enrichment of opportunistic yeasts capable of causing invasive disease.^64^ These disruptions in critically ill patients have been associated with a multitude of negative consequences such the development of ventilator-associated pneumonia and increased re-infection and re-admission rates.^65^^,^^66^

Gut microbiome alteration can disable the protective immunomodulatory effects of commensal microbiota, and decrease the production of beneficial metabolites such as short-chain fatty acids (SCFA).^61^^,^^66^, ^67^, ^68^ Studies utilizing murine sepsis models have shown that obligate anaerobic gut bacteria, which produce butyrate and other SCFAs via the fermentation of otherwise indigestible fibres in the intestine, play a protective role during severe pneumonia as well as abdominal sepsis.^68^^,^^69^ Among the pleiotropic effects of SCFAs is their ability to imprint an antimicrobial program in macrophages inducing the production of antimicrobial peptides by inhibiting histone deacetylase 3.^70^ An altered intestinal microbiome can also have a significant impact on distant organs. For instance, neutrophil homeostasis is regulated by continuous input from commensal Gammaproteobacteria. These bacteria express cell-surface LPS which triggers IL-17A production by innate lymphoid cells via a TLR4-induced signalling cascade, leading to an increase in plasma granulocyte colony-stimulating factor levels. This mechanism plays an essential role in host defence against *E. coli* sepsis in neonatal mice.^71^

Epithelial barrier function can be compromised due to the microbiome disturbances as seen in patients with sepsis. This not only leads to a translocation of pathobionts but also to a disruption of normal antigen sampling and an impairment of the antibody-mediated host defence against invading pathogens. In a preclinical model of polymicrobial sepsis it has been demonstrated that a variety of commensal microbes including several members of the Proteobacteria phylum contribute to a protective increase in serum IgA levels in T cell-dependent fashion.^72^ Of interest, recent insights show that differential mucosal and systemic microbiota exposure can also shape the B cell repertoire.^73^

## Endotypes

The heterogeneity of sepsis likely is an important denominator in the failure of immune modulatory trials in patients with sepsis. Recent investigations have attempted to stratify patients in subgroups that are more homogeneous based on common features of their immune response.^8^ Precision medicine, denoting diagnostic and therapy strategies that take individual patient characteristics into consideration, has not been widely adopted in the field of sepsis. Key in precision medicine are prognostic and predictive enrichment, wherein prognostic enrichment refers to selection of patients with a high likelihood of a relevant disease outcome, and predictive enrichment refers to selection of those who are more likely to react positively to a particular therapy based on a biological mechanism.^8^ While there is consensus that application of both prognostic and predictive enrichment is needed for successful evaluation of sepsis therapies targeting the immune response, the challenge lies in implementation of predictive enrichment, due to a relatively limited understanding of the dominant mechanisms driving the immunopathology of sepsis.^8^ The term endotype has been introduced to indicate a biological subtype defined by distinct pathophysiological mechanisms.^2^^,^^74^ Endotypes are different from what has been named subphenotypes, which indicate a group characterized by a set of features that is not necessarily linked by a common pathophysiological mechanism. Considering the scope of this review we here briefly discuss endotypes only; subphenotypes are reviewed elsewhere.^2^^,^^74^

Several groups reported sepsis endotypes based on whole blood leukocyte gene expression profiles in patients with sepsis. In patients with sepsis caused by community-acquired pneumonia two so-called Sepsis Response Signatures (SRS) were identified: SRS1 and SRS2.^75^ SRS1 was associated with a higher mortality and consistent with an immune suppressive phenotype, with gene expression profiles indicating endotoxin tolerance, HLA class II downregulation and T cell exhaustion.^75^ The SRS1 and SRS2 endotypes were also detected in a retrospective analysis of a clinical trial investigating the effect of corticosteroid therapy in patients with septic shock.^76^ Importantly, corticosteroid therapy appeared associated with harm in the SRS2 endotype and no treatment effect in SRS1 patients, suggesting that the SRS1 and SRS2 subdivision could have relevance for therapeutic decisions.^76^ Our group reported four sepsis transcriptome endotypes, named Mars1 to Mars4, in patients with sepsis.^77^ Mars1 was associated with increased mortality across different cohorts and its gene expression profile was indicative of dampened innate and adaptive immunity. Mars3 had a relatively low mortality risk, and its transcriptome profile pointed at an upregulation of adaptive immunity and increased T cell function.^77^ Comparative analyses revealed overlap between Mars3 and the earlier described SRS2 endotype.^75^^,^^77^ Another investigation reported three subgroups based on blood transcriptomes, termed “inflammopathic” (characterized by innate immune activation and a higher mortality), “adaptive” (adaptive immune activation; lower mortality), and “coagulopathic” (platelet degranulation and coagulation dysfunction; higher mortality and older).^78^ The most recent study in this field, conducted in patients with early sepsis, identified five endotypes, named “neutrophilic-suppressive” (associated with neutrophil activation and immune suppression), “inflammatory” (increased pro-inflammatory response), “innate host defence” (interleukin signalling), “interferon” (increased IFN-α,β,γ) and “adaptive” (activation of a variety of pathways including increased adaptive immunity).^79^ Taken together, these studies indicate that blood leukocyte transcriptomes can be used to divide sepsis patients in different groups with distinct immune profiles and clinical outcomes, and possibly with different responses to specific sepsis therapies. Notably, endotypes generated by unbiased clustering techniques differ across studies and consensus is needed regarding the classification of sepsis patients based on transcriptome data. Other “omics” techniques may also be valuable in stratifying patients into subgroups. For example, three subgroups were identified among patients with early sepsis based on plasma metabolite and lipid profiles; these groups showed different disease severities at presentation associated with distinct clinically relevant outcomes.^80^

## Conclusion

Our better understanding of the pathogenesis of sepsis has thus far not resulted in a specific therapy that targets the immune response. Stratifying patients into more homogeneous groups based on their immune profile and predictive enrichment of study populations are essential tools to improve the likelihood of success in clinical trials. Possibly, for the next sepsis definition, Sepsis-4, sepsis and septic shock will not be defined anymore as a syndrome but rather as a group of distinguishable diseases, each characterized by specific host response aberrations and linked biomarkers.^81^ In line, a recent perspective article on redefining critical illness suggested to de-emphasize the universal use of syndromes and focus on the underlying biological changes that underpin critical illness states—such as sepsis, acute respiratory distress syndrome and acute kidney injury—and that may be amenable to treatment.^82^ Computational methods, such as machine learning may assist in processing large data sets, clinical, physiological and immunological, and detect meaningful patterns that could instruct therapeutic interventions. More attention should be given to the underlying mechanisms of long-term consequences of sepsis, which include cognitive impairments, cardiovascular morbidity and an increased rate of hospital readmissions. Sepsis still is an imprecisely defined syndrome that cannot be treated by specific therapeutics. A major challenge for the coming years will be to translate our increasing knowledge about the molecular mechanisms underlying sepsis into therapies that improve relevant patient outcomes.

## Outstanding questions

**Fig. 3:**
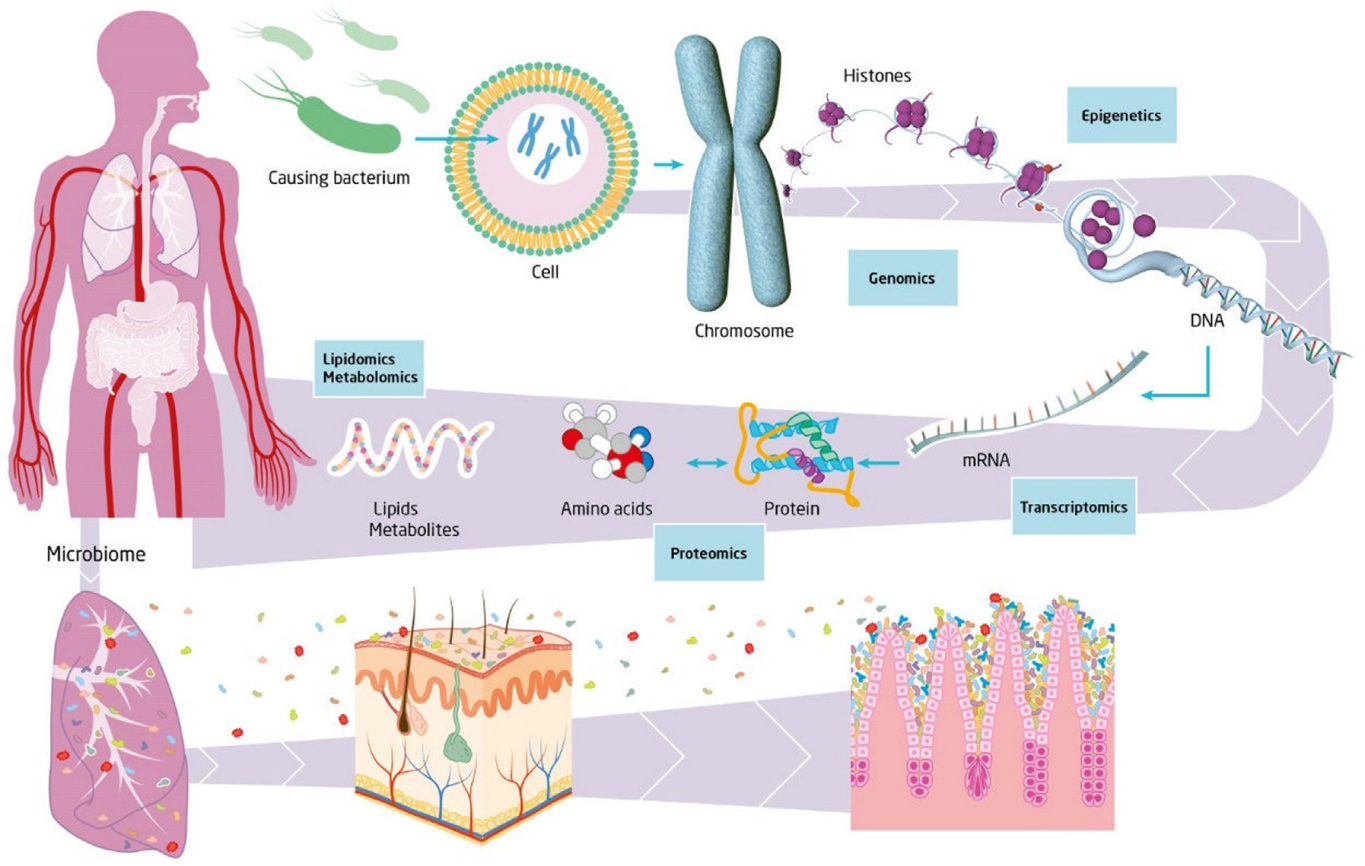
**Deriving biological meaning from multi-omics analysis in sepsis.** The complexity of the septic response is being unravelled following the progress in the major -omics fields of genomics, epigenomics, transcriptomics, metabolomics, proteomics, lipidomics and microbiomics. The next challenge will be to fully integrate these multi-omics approaches in order to derive biologically meaningful insights which will lead to novel clinical applications that will be of value for the patient with sepsis.

Multiple outstanding questions should be addressed in future research. Most of our understanding of sepsis is based on immune function measurements in the systemic compartment. An important question is whether the various immune malfunctions converge at the level of one or several common key molecules or terminal pathways for all immune compartments.^41^ And what are the organ-specific immunological alterations in patients with sepsis caused by different pathogens and how do they develop over time? The advances made in the -omics technologies have helped to further unravel the complexity of sepsis through high-dimensional data analysis^83^ (Fig. 3). However, how will we derive clinical meaningful applications from the integration of all data derived from genomics, epigenomics, transcriptomics, proteomics, lipidomics, and microbiomics etc.?

Our increased understanding of sepsis immunity has led to the identification of novel treatment targets that are being tested in the clinic (Fig. 1). Thus far sepsis treatments evaluated in clinical trials aimed to modulate the hyperinflammatory and/or immunosuppressive features of sepsis. Targeting disease tolerance, reflecting the tissue damage control mechanisms that adjust the metabolic output of host tissues to different forms of stress and damage associated with infection,^84^ might be another effective approach to enable the resolution of sepsis. An ultimate goal is to treat sepsis tailored to the individual based on the causative pathogen and the specific immune response in a time-dependent manner.

## Search strategy and selection criteria

References of this review were collected using PubMed for relevant articles published in 2003–2022, using the terms “sepsis”, “septic shock”, “pathogens”, “pattern recognition receptors”, “toll-like receptors”, “cytokines”, “neutrophil extracellular traps”, “immune suppression”, “coagulation”, “complement”, “endotypes”, and “gut microbiome”.

## Contributors

WJW and TvdP conceived and wrote the manuscript. The authors read and approved the final version of the manuscript.

## Data sharing statement

Not applicable as this is a review article.

## Declaration of interests

The authors declare no conflict of interest.
